# Diversity, evolution, and emergence of fish viruses

**DOI:** 10.1128/jvi.00118-24

**Published:** 2024-05-24

**Authors:** Vincenzo A. Costa, Edward C. Holmes

**Affiliations:** 1 Sydney Institute for Infectious Diseases, School of Medical Sciences, The University of Sydney, Sydney, New South Wales, Australia; Department of Microbiology, New York University, New York, New York, USA

**Keywords:** virus, virome, fish, emerging disease, aquaculture, ecology, evolution

## Abstract

The production of aquatic animals has more than doubled over the last 50 years and is anticipated to continually increase. While fish are recognized as a valuable and sustainable source of nutrition, particularly in the context of human population growth and climate change, the rapid expansion of aquaculture coincides with the emergence of highly pathogenic viruses that often spread globally through aquacultural practices. Here, we provide an overview of the fish virome and its relevance for disease emergence, with a focus on the insights gained through metagenomic sequencing, noting potential areas for future study. In particular, we describe the diversity and evolution of fish viruses, for which the majority have no known disease associations, and demonstrate how viruses emerge in fish populations, most notably at an expanding domestic-wild interface. We also show how wild fish are a powerful and tractable model system to study virus ecology and evolution more broadly and can be used to identify the major factors that shape vertebrate viromes. Central to this is a process of virus-host co-divergence that proceeds over many millions of years, combined with ongoing cross-species virus transmission.

## INTRODUCTION

The advent of total RNA sequencing (i.e., metatranscriptomics) has revolutionized our ability to document the animal virosphere. This includes studies of the most ecologically diverse and speciose group of vertebrates—fish. Recent metatranscriptomic studies have shown that fish naturally carry a diverse array of viruses, including distant relatives of notable human pathogens such as SARS-CoV-2, hepatitis C virus, influenza viruses, filoviruses, and paramyxoviruses, as well as a variety of DNA viruses including parvoviruses, papillomaviruses, and hepadnaviruses (1–10). However, despite the ongoing characterization of an ever-growing number of fish viruses, we lack a detailed understanding of virus evolution, ecology, and emergence in aquatic ecosystems and for hosts that have evolutionary histories spanning more than 500 million years.

The importance of understanding the fish virome is given social context by the fact that the production and consumption of seafood has doubled over the last 50 years and is expected to rise exponentially (11). In 2020, global production of seafood was over 178 million tons, with an estimated value of 406 billion US dollars (11). Fisheries and aquaculture provide half of the world’s population with approximately 20% of their animal protein intake, offering essential omega-3 fatty acids, vitamins, and minerals while generating significantly low greenhouse-gas emissions (11, 12). This makes fish a sustainable and cost-effective system in the face of human population growth and climate change. Yet despite the global significance of aquaculture, its expansion has led to the emergence of highly pathogenic viruses that often go understudied.

Emerging viral diseases occur when a virus gains the ability to infect a novel host following cross-species transmission (i.e., host jumping). Viruses must initially overcome a variety of host molecular barriers to complete their replication cycle, such as receptor binding, genome replication, and antiviral immune responses (13). Subsequently, viruses must possess (or evolve) the ability to be transmitted between conspecifics and subsequently establish and sustain transmission chains for successful emergence (13). While the initial stages predominantly involve molecular processes, interactions that occur both between and within host species will determine whether such cross-species transmission events leads to dead-end (i.e., “spill-over”) or sustained (i.e., epidemic/epizootic) onward infections in the new host species (13, 14). Importantly, these interactions are often exacerbated by anthropogenic processes, including the direct (i.e., deliberate) and indirect (e.g., species range shifts as a result of climate change) movement of hosts over vast geographic distances. Hence, viral emergence involves interactions between ecological and evolutionary factors that ultimately facilitate the transmission of viruses from a donor (original) to recipient (novel) host species.

Aquaculture itself is an important driver of emerging viral diseases: viruses are often translocated to new geographic areas through the international trade of aquatic animals and there is a high level of connectivity at the wild-domestic interface that exposes viruses to naïve, dense fish monocultures. Globally, emerging infectious diseases in fish are responsible for annual economic losses of up to six billion US dollars (15). Indeed, the World Organisation for Animal Health (WOAH) currently lists 11 notifiable diseases of fish, with 9 of these caused by viruses (16). Additionally, other viruses, including reoviruses, flaviviruses, hantaviruses, picornaviruses, caliciviruses iridoviruses, and parvoviruses, have recently led to disease outbreaks in both wild and domestic ecosystems (17–22). These outbreaks have also affected endangered species, pushing them closer to the brink of extinction (23). The often-severe impact of emerging viral diseases makes it imperative to understand the composition of viruses that circulate in aquatic ecosystems, as well as the ecological and evolutionary processes that facilitate cross-species transmission and disease emergence.

## DIVERSITY AND EVOLUTION OF FISH VIRUSES

**Fig 1:**
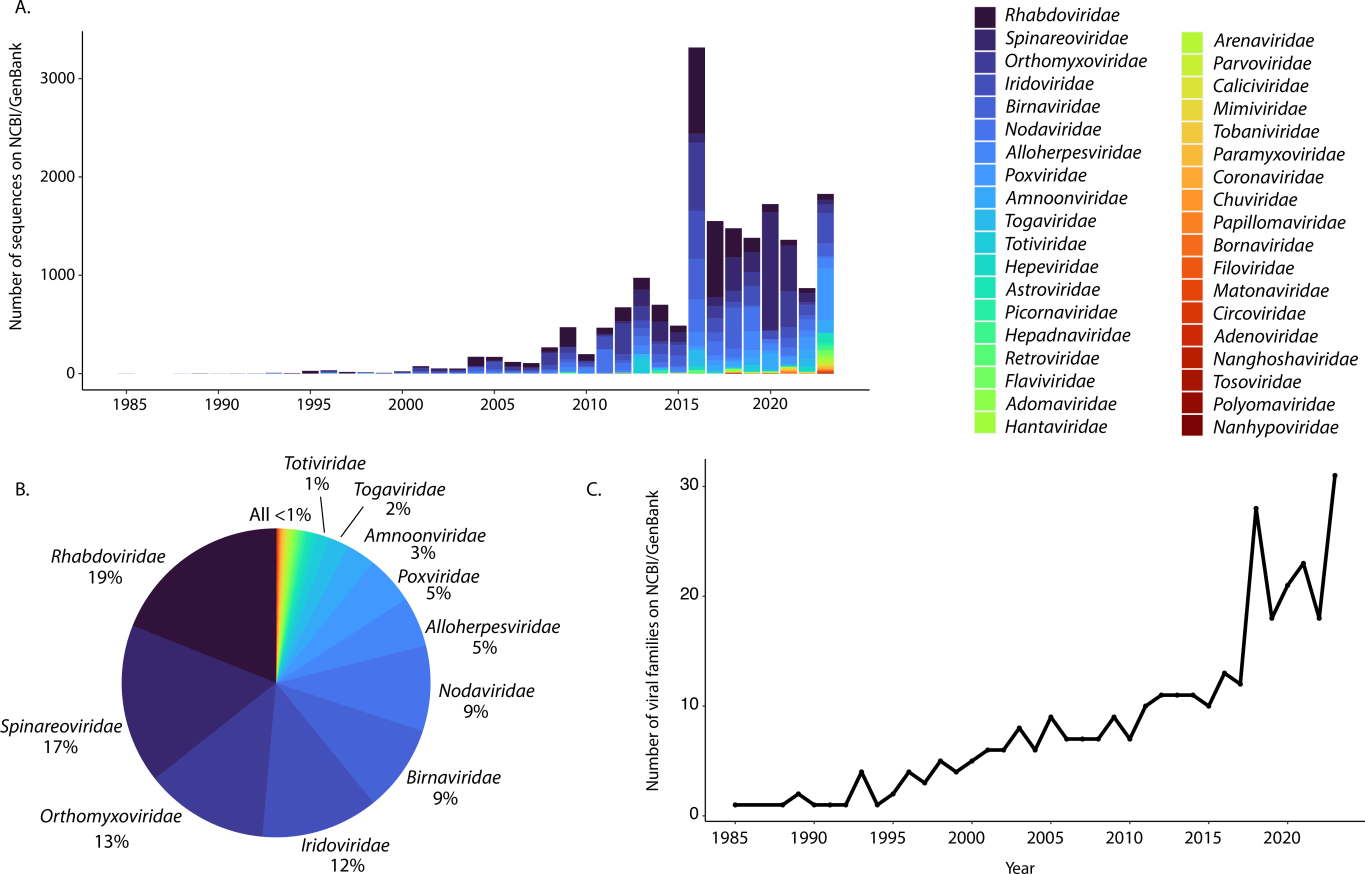
Viral families known to infect fish. (**A**) Viral sequences on NCBI/GenBank by year of release. (**B**) Percentage of viral sequences for fish viral families on NCBI/GenBank. (**C**) Number of viral families on NCBI/GenBank by year of release.

A major portion of our current understanding of the diversity of fish viruses is derived from a limited set of virus families. Indeed, members of the *Rhabdoviridae, Spinareoviridae*, *Orthomyxoviridae*, *Iridoviridae*, *Birnaviridae*, and *Nodaviridae* collectively account for some 80% of the nucleotide sequences available on NCBI/GenBank (Fig. 1B). These viral families contain important pathogens of fish, many of which are listed as notifiable by the WOAH (Table 1). However, the global fish virome is evidently far larger and more diverse. A simple indication is that a single large-scale metatranscriptomic study of vertebrates increased the number of families of fish viruses on NCBI/GenBank by ~58% (Fig. 1C) (1), and nearly all viral families known to infect humans have now been identified in fish (Fig. 1A). Clearly, fish naturally carry a diverse array of viruses that do not cause overt disease, many of which are related to pathogenic viruses. We now discuss these viral groups in turn.

**TABLE 1:** Notable viral pathogens of fish^*a*^

Viral species	Viral genus	Viral family	Type	Susceptible fish families	Notifiable disease (WOAH)	References
*Guitarfish adomavirus*	Unclassified	*Adomaviridae*	dsDNA	*Rhinidae*	No	24
*American eel adomavirus*	Unclassified	*Adomaviridae*	dsDNA	*Anguillidae*	No	25
*Micropterus dolomieu adomavirus 2*	Unclassified	*Adomaviridae*	dsDNA	*Centrarchidae*	No	26
*Marbled eel adomavirus*	Unclassified	*Adomaviridae*	dsDNA	*Anguillidae*	No	27
*Japanese eel adomavirus*	Unclassified	*Adomaviridae*	dsDNA	*Anguillidae*	No	28
*Anguillid herpesvirus 1*	*Cyprinivirus*	*Alloherpesviridae*	dsDNA	*Anguillidae*	No	29
*Cyprinid herpesvirus 1*	*Cyprinivirus*	*Alloherpesviridae*	dsDNA	*Cyprinidae*	No	30
*Cyprinid herpesvirus 2*	*Cyprinivirus*	*Alloherpesviridae*	dsDNA	*Cyprinidae*	No	31
*Cyprinid herpesvirus 3*	*Cyprinivirus*	*Alloherpesviridae*	dsDNA	*Cyprinidae*	Yes	32
*Acipenserid herpesvirus 2*	*Ictalurivirus*	*Alloherpesviridae*	dsDNA	*Acipenseridae*	No	33
*Ictalurid herpesvirus 1*	*Ictalurivirus*	*Alloherpesviridae*	dsDNA	*Ictaluridae*	No	34
*Ictalurid herpesvirus 2*	*Ictalurivirus*	*Alloherpesviridae*	dsDNA	*Ictaluridae*	No	35
*Salmonid herpesvirus 1*	*Salmonivirus*	*Alloherpesviridae*	dsDNA	*Salmonidae*	No	36
*Salmonid herpesvirus 2*	*Salmonivirus*	*Alloherpesviridae*	dsDNA	*Salmonidae*	No	37
*Salmonid herpesvirus 3*	*Salmonivirus*	*Alloherpesviridae*	dsDNA	*Salmonidae*	No	38
*Atlantic cod herpesvirus*	Unclassified	*Alloherpesviridae*	dsDNA	*Gadidae*	No	39
*Pilchard herpesvirus*	Unclassified	*Alloherpesviridae*	dsDNA	*Clupeidae*	No	40
*Tilapia herpesvirus*	Unclassified	*Alloherpesviridae*	dsDNA	*Cichlidae*	No	41
*Acipenserid herpesvirus 1*	Unclassified	*Alloherpesviridae*	dsDNA	*Acipenseridae*	No	42
*Esocid herpesvirus 1*	Unclassified	*Alloherpesviridae*	dsDNA	*Esocidae*	No	43
*Percid herpesvirus 2*	Unclassified	*Alloherpesviridae*	dsDNA	*Percidae*	No	44
*Silurid herpesvirus 1*	Unclassified	*Alloherpesviridae*	dsDNA	*Siluridae*	No	45
*Tilapia lake virus*	*Tilapinevirus*	*Amnoonviridae*	+ssRNA	*Cichlidae, Cyprinidae, Osphronemidae*	Yes	46
*Infectious pancreatic necrosis virus (IPNV*)	*Aquabirnavirus*	*Birnaviridae*	dsRNA	*Salmonidae, Scophthalmidae, Anguillidae, Pleuronectidae, Gadidae, Sparidae, Cichlidae*	No	47, 48
*Yellowtail ascites virus*	*Aquabirnavirus*	*Birnaviridae*	dsRNA	*Carangidae*	No	49
*Lates calcarifer birnavirus*	*Blosnavirus*	*Birnaviridae*	dsRNA	*Latidae*	No	50
*Largemouth bass birnavirus*	Unclassified	*Birnaviridae*	dsRNA	*Centrarchidae*	No	18
*Atlantic salmon calicivirus*	*Salovirus*	*Caliciviridae*	+ssRNA	*Salmonidae*	No	51
*Fathead minnow calicivirus*	*Minovirus*	*Caliciviridae*	+ssRNA	*Cyprinidae*	No	52
*Yellow catfish calicivirus*	Unclassified	*Unclassified Picornavirales*	+ssRNA	*Ictaluridae*	No	20
*Cyclopterus lumpus virus*	Unclassified	*Flaviviridae*	+ssRNA	*Cyclopteridae*	No	17
*Salmon flavivirus*	Unclassified	*Flaviviridae*	+ssRNA	*Salmonidae*	No	22
*Perch actinovirus*	Unclassified	*Hantaviridae*	−ssRNA	*Percidae*	No	19
*Santee-Cooper ranavirus*	*Ranavirus*	*Iridoviridae*	dsDNA	*Centrarchidae, Sinipercidae, Labridae, Cyprinidae*	No	6, 53–57
*Epizootic hematopoietic necrosis virus (EHNV*)	*Ranavirus*	*Iridoviridae*	dsDNA	*Percidae*	Yes	58
*European catfish virus*	*Ranavirus*	*Iridoviridae*	dsDNA	*Siluridae*	No	59
*Singapore grouper iridovirus*	*Ranavirus*	*Iridoviridae*	dsDNA	*Serranidae*	No	60
*Infectious spleen and kidney necrosis virus (ISKNV*)	*Megalocytivirus*	*Iridoviridae*	dsDNA	*Sparidae, Carangidae, Scombridae, Scaridae, Oplegnathidae, Rachycentridae, Haemulidae, Lethrinidae, Kyphosidae, Scorpaenidae, Sciaenidae, Serranidae, Latidae, Moronidae, Centrarchidae, Paralichthyidae, Tetraodontidae, Mugillidae, Sinipercidae, Apogonidae, Arapaimidae, Cichlidae, Characidae, Characidae, Cobitidae, Ephippidae, Helostomatidae, Hemiodontidae, Loricariidae, Labridae, Nothobranchiidae, Osphronemidae, Poeciliidae, Pomacanthidae*	Yes	61 – 63
*European chub iridovirus*	*Megalocytivirus*	*Iridoviridae*	dsDNA	*Cyprinidae*	No	64
*Scale drop disease virus*	*Megalocytivirus*	*Iridoviridae*	dsDNA	*Latidae*	No	65
*Lymphocystis disease virus 1*	*Lymphocystivirus*	*Iridoviridae*	dsDNA	*Pleuronectidae*	No	66
*Lymphocystis disease virus 2*	*Lymphocystivirus*	*Iridoviridae*	dsDNA	*Pleuronectidae*	No	67
*Lymphocystis disease virus 3*	*Lymphocystivirus*	*Iridoviridae*	dsDNA	*Sparidae*	No	68
*Lymphocystis disease virus 4*	*Lymphocystivirus*	*Iridoviridae*	dsDNA	*Sciaenidae*	No	69
*Namao virus*	Unclassified	*Mimiviridae*	dsDNA	*Acipenseridae*	No	70
*White Sturgeon Iridovirus*	Unclassified	*Mimiviridae*	dsDNA	*Acipenseridae*	No	70
*Acipenser Iridovirus-European*	Unclassified	*Mimiviridae*	dsDNA	*Acipenseridae*	No	70
*Shortnose sturgeon virus*	Unclassified	*Mimiviridae*	dsDNA	*Acipenseridae*	No	70
*Missouri River sturgeon iridovirus*	Unclassified	*Mimiviridae*	dsDNA	*Acipenseridae*	No	70
*British Columbia white sturgeon virus*	Unclassified	*Mimiviridae*	dsDNA	*Acipenseridae*	No	70
*Barfin flounder nervous necrosis virus (BFNNV*)	*Betanodavirus*	*Nodaviridae*	+ssRNA	*Pleuronectidae, Gadidae, Soleidae, Labridae*	No	71
*Redspotted grouper nervous necrosis virus (RGNNV*)	*Betanodavirus*	*Nodaviridae*	+ssRNA	*Oplegnathidae, Mugilidae, Latidae, Ephippidae, Carangidae, Lutjanidae, Moronidae, Pomacentridae, Rachycentridae, Sciaenidae, Scombridae, Serranidae, Paralicthyidae, Sebastidae, Engraulidae, Lateolabracidae, Mullidae, Polycentridae, Serrasalmidae, Siganidae, Sparidae, Stromateidae, Synanceiidae, Tetraodontidae, Anguillidae, Poeciliidae, Blenniidae, Centrarchidae, Cichlidae, Eleotridae, Percidae, Siluridae, Congridae, Atherinidae, Synodontidae, Bathrachoididae, Belonidae, Exocoetidae, Latridae, Clupeidae, Dussumieriidae, Gadidae, Merluciidae, Heterodontidae, Apogonidae, Centrolophidae, Gobiidae, Haemulidae, Kyphosidae, Labridae, Leiognathidae, Nemipteridae, Percophidae, Priacanthidae, Sphyraenidae, Terapontidae, Zanclidae, Citharidae, Plotosidae, Scorpaenidae, Triglidae, Fistuliridae, Diodontidae, Monacanthidae*	No	71
*Striped jack nervous necrosis virus (SJNNV*)	*Betanodavirus*	*Nodaviridae*	+ssRNA	*Carangidae, Soleidae, Anguillidae, Sciaenidae, Scombridae*	No	71
*Tiger puffer nervous necrosis virus (TPNNV*)	*Betanodavirus*	*Nodaviridae*	+ssRNA	*Tetraodontidae*	No	71
*RGNNV/SJNNV (reassortant*)	*Betanodavirus*	*Nodaviridae*	+ssRNA	*Moronidae, Sparidae, Soleidae, Carangidae,*	No	71
*Infectious salmon anemia virus*	*Isavirus*	*Orthomyxoviridae*	−ssRNA	*Salmonidae*	Yes	61
*Pilchard orthomyxovirus*	Unclassified	*Orthomyxoviridae*	−ssRNA	*Clupeidae, Salmonidae*	No	72
*Sparus aurata papillomavirus*	Unclassified	*Papillomaviridae*	dsDNA	*Sparidae*	No	68
*Wels catfish papillomavirus*	Unclassified	*Papillomaviridae*	dsDNA	*Siluridae*	No	73
*Atlantic salmon aquaparamyxovirus*	*Aquaparamyxovirus*	*Paramyxoviridae*	−ssRNA	*Salmonidae*	No	74
*Tilapia parvovirus*	Unclassified	*Parvoviridae*	ssDNA	*Cichlidae*	No	10
*Bluegill picornavirus*	*Limnipivirus*	*Picornaviridae*	+ssRNA	*Centrarchidae*	No	75
*Eel picornavirus*	*Potamipivirus*	*Picornaviridae*	+ssRNA	*Anguillidae*	No	76
*Glithead seabream picornavirus*	*Potamipivirus*	*Picornaviridae*	+ssRNA	*Sparidae*	No	77
*Clownfish picornavirus*	*Limnipivirus*	*Picornaviridae*	+ssRNA	*Pomacentridae*	No	21
*Carp edema virus*	*Unclassified*	*Poxviridae*	dsDNA	*Cyprinidae*	No	78
*Salmon gillpox virus*	*Salmonpoxvirus*	*Poxviridae*	dsDNA	*Salmonidae*	No	7
*Walleye dermal sarcoma virus (WDSV*)	*Epsilontretrovirus*	*Retroviridae*	RT	*Percidae*	No	79
*Walleye epidermal hyperplasia virus 1*	*Epsilontretrovirus*	*Retroviridae*	RT	*Percidae*	No	80
*Walleye epidermal hyperplasia virus 2*	*Epsilontretrovirus*	*Retroviridae*	RT	*Percidae*	No	80
*Atlantic salmon swim bladder sarcoma virus (SSSV*)	*Epsilontretrovirus*	*Retroviridae*	RT	*Salmonidae*	No	81
*Viral haemorrhagic septicaemia virus* (*VHSV*)	*Novirhabdovirus*	*Rhabdoviridae*	−ssRNA	*Ammodytidae, Carangidae, Centrarchidae, Clupeidae, Cyclopteridae, Cyprinidae, Embiotocidae, Engraulidae, Esocidae, Fundulidae, Gadidae, Gasterosteidae, Gobiidae, Ictaluridae, Labridae, Lotidae, Moronidae, Mullidae, Osmeridae, Paralichthyidae, Percidae, Petromyzontidae, Pleuronectidae, Rajidae, Salmonidae, Scophthalmidae, Sciaenidae, Scombridae, Soleidae, Uranoscopidae*	Yes	61, 82
*Infectious hematopoietic necrosis virus (IHNV*)	*Novirhabdovirus*	*Rhabdoviridae*	−ssRNA	*Salmonidae*	Yes	61, 82
*Hirame rhabdovirus*	*Novirhabdovirus*	*Rhabdoviridae*	−ssRNA	*Paralichthyidae*	No	82
*Snakehead rhabdovirus*	*Novirhabdovirus*	*Rhabdoviridae*	−ssRNA	*Channidae*	No	82
*Spring viraemia of carp virus (SVCV*)	*Sprivivirus*	*Rhabdoviridae*	−ssRNA	*Cyprinidae*	Yes	82
*Pike fry rhabdovirus*	*Sprivivirus*	*Rhabdoviridae*	−ssRNA	*Esocidae*	No	82
*Perhabdovirus perca*	*Perhabdovirus*	*Rhabdoviridae*	−ssRNA	*Percidae, Salmonidae*	No	82
*Perhabdovirus trutta*	*Perhabdovirus*	*Rhabdoviridae*	−ssRNA	*Salmonidae*	No	82
*Perhabdovirus anguilla*	*Perhabdovirus*	*Rhabdoviridae*	−ssRNA	*Anguillidae*	No	82
*Perhabdovirus leman*	*Perhabdovirus*	*Rhabdoviridae*	−ssRNA	*Percidae*	No	82
*Siniperhavirus zoarces*	*Siniperhavirus*	*Rhabdoviridae*	−ssRNA	*Zoarcidae*	No	82, 83
*Siniperhavirus chuatsi*	*Siniperhavirus*	*Rhabdoviridae*	−ssRNA	*Sinipercidae, Anguillidae, Channidae, Centrarchidae*	No	82
*Scophthalmus maximus rhabdovirus*	*Scophrhavirus*	*Rhabdoviridae*	−ssRNA	*Scophthalmidae*	No	82
*Aquareovirus A*	*Aquareovirus*	*Spinareoviridae*	dsRNA	*Salmonidae, Centrarchidae*	No	84
*Aquareovirus B*	*Aquareovirus*	*Spinareoviridae*	dsRNA	*Salmonidae*	No	84
*Aquareovirus C*	*Aquareovirus*	*Spinareoviridae*	dsRNA	*Cyprinidae*	No	85
*Aquareovirus D*	*Aquareovirus*	*Spinareoviridae*	dsRNA	*Ictaluridae*	No	86
*Aquareovirus E*	*Aquareovirus*	*Spinareoviridae*	dsRNA	*Scophthalmidae*	No	87
*Aquareovirus F*	*Aquareovirus*	*Spinareoviridae*	dsRNA	*Salmonidae*	No	88
*Aquareovirus G*	*Aquareovirus*	*Spinareoviridae*	dsRNA	*Cyprinidae*	No	89
*Largemouth bass reovirus*	*Orthoreovirus*	*Spinareoviridae*	dsRNA	*Centrarchidae*	No	90
*Piscine orthoreovirus (PRV*)	*Orthoreovirus*	*Spinareoviridae*	dsRNA	*Salmonidae*	No	23
*Fathead minnow nidovirus*	*Bafinivirus*	*Tobaniviridae*	+ssRNA	*Cyprinidae, Esocidae*	No	91, 92
*Chinook salmon bafinivirus*	*Bafinivirus*	*Tobaniviridae*	+ssRNA	*Salmonidae, Cyprinidae, Siluridae, Paralichthyidae*	No	93 – 95
*Trout granulomatous virus*	Unclassified	*Tobaniviridae*	+ssRNA	*Salmonidae*	No	96
*Salmonid alphavirus*	*Alphavirus*	*Togaviridae*	+ssRNA	*Salmonidae, Pleuronectidae*	Yes	97
*Piscine myocarditis virus*	Unclassified	*Totiviridae*	dsRNA	*Salmonidae*	No	98

^*a*^ dsDNA, double-stranded DNA; ssDNA, single-stranded DNA; +ssRNA, positive-sense single-stranded RNA; −ssRNA, negative-sense single-stranded RNA; dsRNA, double-stranded RNA; RT, reverse transcribing.

### Notable RNA viral pathogens of fish

#### Rhabdoviridae

Rhabdoviruses are important pathogens of fish and have been detected in over 34 different families across all fish classes: Agnatha (jawless fishes), Chondrichthyes (cartilaginous fishes), and Osteichthyes (bony fishes) (Table 1) (61). A large portion of this diversity stems from the genus *Novirhabdovirus* (subfamily *Gammarhabdovirinae*) that exclusively infects fish and includes *Viral haemorrhagic septicaemia virus* (VHSV), *Infectious hematopoietic necrosis virus* (IHNV), and *Hirame rhabdovirus* and *Snakehead rhabdovirus* (82). Almost all members of the *Rhabdoviridae* contain five structural genes: nucleoprotein, phosphoprotein, matrix protein, glycoprotein, and the RNA-dependent RNA polymerase (L) (82). The novirhabdoviruses contain an additional nonvirion (NV) gene that suppresses apoptosis during the early stages of infection (99). The NV gene experiences the highest substitution (i.e., evolutionary) rate among VHSV genes, and single amino acid changes have been associated with increases in virulence (100, 101).

VHSV is known to infect over 70 fish species from 30 families (61). Notably, it only occurs in the Northern Hemisphere and has one of the broadest host ranges among vertebrate viruses, making it a significant threat to wild and farmed fish populations. Phylogenetic analysis of the nucleoprotein and glycoprotein has classified VSHV into four genotypes that broadly correspond to host range and geography (61, 99). Genotype I incorporates five subtypes (a–e), nearly all of which are associated with disease outbreaks in rainbow trout (61). Genotype II has been primarily detected in wild herring and lamprey from the Baltic Sea, while genotype III infects a variety of wild and farmed fishes in the North Sea, including herring, cod as well as an outbreak in cleaner wrasses at an aquaculture facility in Scotland (102, 103). Genotype IV exhibits a broad host and geographic range, infecting over 39 different species in North America, Asia, and Europe (61).

Among other novirhabdoviruses, IHNV exhibits a much narrower host range, occurring primarily in salmonids (61, 82), although it has also been shown to replicate in asymptomatic pike which may act as natural reservoirs (104). IHNV emerged during the 1980s, where it is now enzootic in rivers throughout North America (105). Following its discovery, IHNV spread to Europe and Asia via contaminated fish eggs (106). Phylogenetic comparisons of the viral glycoprotein have classified IHNV into five genogroups (denoted U, M, L, E, J). Of these, genogroup E occurs in Europe, Asia, Africa, and USA, with all other genogroups currently restricted to the USA (61, 82).

Other pathogenic rhabdoviruses belong to the *Perhabdovirus* and *Sprivivirus*, *Siniperhavirus*, and *Scoprhavirus* genera, which collectively form part of the *Alpharhabdovirinae* subfamily. Perhabdoviruses were first detected in the 1970s from diseased eels in Europe and the USA (now classified as *Perhabdovirus anguilla*) and then later discovered in a variety of perch species (*Perhabdovirus perca* and *Perhabdovirus leman*) and sea trout (*Perhabdovirus trutta*) during the 1980s and 1990s (82, 107). During the early 2000s, *Siniperhavirus chuatsi* emerged in diseased mandarin fish (*Siniperca chuatsi*) in China, forming a new genus, *Siniperhavirus* (108). *Siniperhavirus chuatsi* has also been isolated from snakeheads, Chinese rice-field eel, and largemouth bass (109). In addition, a novel *Siniperhavirus* species, *Siniperhavirus zoarces*, was the causative agent of two disease outbreaks in eelpouts along the Swedish coastline in the Baltic Sea (83).

#### Spinareoviridae

Aquareoviruses are the most diverse group of reoviruses that infect fish and were first identified in moribund golden shiners in 1977 (86). Subsequently, during the 1980s, related viruses were identified in diseased chum salmon and channel catfish, and most notably in farmed grass carp with mortality rates of 85% (84–86). The genus *Aquareovirus* is arranged into seven member species (A–G), with several others awaiting classification (110) (Table 1). While most aquareoviruses cause mild disease, grass carp reovirus (*Aquareovirus C*) causes severe haemorrhagic disease in farmed cyprinids (111). Phylogenetic analysis of the VP2 gene (the RNA-dependent RNA polymerase; RdRp) has revealed three genotypes of grass carp reovirus that are widely distributed among aquaculture facilities in China (111).

During 1999, heart and skeletal muscle inflammation (HSMI) emerged in farmed Atlantic Salmon in Norway (112), although the causative agent, *Piscine orthoreovirus* (PRV), was not identified until 2010 (113). PRV is a member of the genus *Orthoreovirus* that also includes viruses of mammals, birds, and reptiles (110). PRV is arranged into three subtypes: PRV-1 occurs in the UK, Ireland, Sweden, Faroe Islands, Iceland, Denmark, France, Germany, Norway, Chile, the USA, and Canada; PRV-2 in Japan; and PRV-3, Chile and Norway (23). Among the subtypes, PRV-1 causes HSMI and primarily infects farmed and wild salmonids including critically endangered populations such as Chinook salmon (23).

#### *Articulavirales*: *Orthomyxoviridae* and *Amnoonviridae*

The RNA virus order *Articulavirales* contains important pathogens of vertebrates including the influenza viruses (*Orthomyxoviridae*), infectious salmon anaemia virus (ISAV) (*Orthomyxoviridae*), and tilapia lake virus (*Amnoonviridae*). ISAV is an important pathogen of sea-farmed Atlantic salmon (*Salmo salar*), causing severe disease outbreaks characterized by hemorrhaging, necrosis, and anemia (61). It was first detected in Norway during the 1980s, then later in Canada, Scotland, Faroe Islands, the USA, and Chile during the 1990s and early 2000s (114, 115). Its genome is arranged into eight segments, including segment 5 (fusion protein) and 6 (hemagglutinin-esterase [HE]) that encode the surface glycoproteins. Together, these proteins play a key role in pathogenicity and virulence (116, 117). The HE protein exhibits receptor binding activity as well as receptor-destroying properties (117). Deletions in the highly polymorphic region (HPR) in the HE protein led to the formation of the highly virulent strain, “HPR-deleted,” associated with systemic disease (116). In contrast, the HPR0 strain, that contains the full-length HPR, causes subclinical infections in Atlantic salmon that are typically detected in gill tissue (118). ISAV has also been detected in Coho salmon in Chile and rainbow trout in Ireland although natural disease outbreaks have only been detected in sea-reared Atlantic salmon (114).

Another orthomyxovirus of concern is pilchard orthomyxovirus (POMV) that was associated with a mass mortality event in wild pilchards (*Sardinops sagax*) in South Australia during 1995 (119). In 2012, POMV was also isolated from diseased Atlantic salmon from various farms across Southern Tasmania (72).

In 2009, high mortality rates among tilapia were observed in fish farms across Israel (120). However, in 2014, the etiology of such mass mortalities was identified as due to an “orthomyxo-like” virus, now known as *Tilapia lake virus* (TiLV), a member of the family *Amnoonviridae* (120). Since its discovery, TiLV has been identified in Colombia, Egypt, India, Indonesia, Malaysia, Peru, Thailand, the Philippines, Tanzania, Uganda, Mexico, and the USA (46). While tilapia are severely affected by TiLV with high rates of mortality, natural infections have been detected in giant gourami (*Osphronemus goramy*), indicating that these species could act as vectors (46).

#### Totiviridae

Cardiomyopathy syndrome (CMS) was first recorded in Atlantic salmon in Norway in 1985, then later spread to the Faroe Islands, Scotland, and Canada by the early 2000s (98). However, the etiological agent, *Piscine myocarditis virus* (PMCV), a member of the *Totiviridae*, was not identified until 2011 (98). Although totiviruses were traditionally known to infect fungi and protozoans, they have now been detected in several fish species such as lumpfishes, carp, bluegill, and shiners (121). While these additional fish viruses fall within the same phylogenetic group as PMCV, they have not been cultured in fish cell lines or associated with disease (121).

#### Birnaviridae

*Infectious pancreatic necrosis virus* (IPNV) was the first fish virus isolated and is a common disease of salmonids, particularly juveniles of rainbow trout and Atlantic salmon (48). IPNV causes infectious pancreatic necrosis—a disease characterized by darkening of the skin, petechial hemorrhaging, abdominal swelling, and most notably, abnormal corkscrew swimming and anorexia (48). IPNV forms part of the genus *Aquabirnavirus* that also includes *Yellowtail ascites virus* that causes ascites and deformity in yellowtail populations (*Seriola quinqueradiata*) in Japan (49). The genus *Blosnavirus* comprises other fish birnaviruses, including *Lates calcarifer birnavirus*, that was associated with disease outbreaks in farmed Asian seabass (*L. calcarifer*) in Singapore (50). In addition, a closely related virus—Largemouth bass birnavirus—was isolated from farmed largemouth bass fingerlings associated with a large disease outbreak in China (18). Despite the divergence between IPNV and blosnaviruses, which are more closely related to the avibirnaviruses that infect birds, Largemouth bass birnavirus exhibits highly similar clinical signs to IPNV, including irregular swimming behavior (18).

#### Togaviridae

The *Togaviridae* contains a single genus, *Alphavirus*, that largely comprises mosquito-borne pathogens including chikungunya virus and eastern equine encephalitis virus (122). Salmonid alphavirus (SAV) is the only member of the *Togaviridae* that infects fish, causing pancreas disease and sleeping disease in Atlantic salmon and rainbow trout (97). SAV is arranged into six genotypes (SAV1-6), all of which cause pancreatic disease (123). Among these genotypes, SAV2 contains both freshwater and marine variants, with the former responsible for sleeping disease in rainbow trout (123). In addition to salmonids, SAV has been detected in several flatfish species (Pleuronectidae) (97).

#### Nodaviridae

Nervous necrosis virus (NNV), the etiological agent of viral nervous necrosis, has caused disease outbreaks in over 56 marine fishes and 13 freshwater fish species worldwide (71). It was first described during the 1980s, associated with larval barramundi from Australia with neurological disease (124). NNV is classified within the genus *Betanodavirus* with four member species—*Barfin flounder nervous necrosis viru*s (BFNNV), *Redspotted grouper nervous necrosis virus* (RGNNV), *Striped jack nervous necrosis virus* (SJNNV), *Tiger puffer nervous necrosis virus* (TPNNV)—as well as a reassortant virus RGNNV/SJNNV (or SJNNV/RGNNV) (71, 125). The optimal growth temperature varies among virus species, which, in turn, affects their geographic distribution. For instance, BFNVV replicates effectively at 15–20°C and infects cold-water fishes (e.g., Atlantic cod) in Northern Europe, America and Japan, while RGNNV affects tropical and temperate fishes with an optimal growth temperature range of 25–30°C (71). RGNNV is the most widespread and is capable of infecting over 57 different fish families that majority of which have been detected without obvious signs of disease (71).

### Notable DNA viral pathogens of fish

#### Iridoviridae

Iridoviruses are among the most widespread and pathogenic group of DNA viruses that infect fish. The *Iridoviridae* are classified into two subfamilies, the *Alphairidovirinae* that exclusively infect ectothermic vertebrates and the *Betairidovirinae* that are associated with both invertebrates and fish (Fig. 3) (62). The *Alphairidovirinae* contain three genera—*Ranavirus, Megalocytivirus,* and *Lymphocystivirus*—that cause severe disease. Notable among the ranaviruses is *Epizootic hematopoietic necrosis virus* that is currently restricted to Australia, where it has caused large disease outbreaks in wild redfin perch (126). The genus *Megalocytivirus* comprises two viral species, *Infectious spleen and kidney necrosis virus* (ISKV) and *Scale drop disease virus*. Owing to their notably wide host range, systemic disease, and asymptomatic infections in a variety of ornamental fishes, ISKNV poses a significant threat to wild and farmed fish populations. ISKNV contains several variants, including red seabream iridovirus, that is known to infect over 40 different species of marine and freshwater fishes (61). Disease outbreaks of ISKNV have been detected primarily in south-east Asia and Japan but have also been detected in imported ornamental fishes in Australia and the USA (63).

#### Alloherpesviridae

The *Alloherpesviridae* are a family of large double-stranded viruses that infect fish and amphibians. Almost all members of the fish-infecting genera, *Cyprinivirus, Salmonivirus, Ictalurivirus*, have been associated with disease outbreaks (127). Notable among the *Alloherpesviridae* is *Cyprinid herpesvirus-3* (CyHV-3), also known as koi herpesvirus, that has caused severe disease in common carp in Europe, the Middle East, Africa, Asia, and North America (61). In 2018, CyHV-3 was responsible for a disease outbreak in more than 2.3 million wild and domestic carp in Iraq, with a mortality rate of 99.42% (128). Moreover, CyHV-3 only affects subspecies of common carp and hybrids including *Cyprinus carpio × Carassius auratus* (61).

#### Poxviridae

Fish poxviruses fall within the subfamily *Choropoxvirinae* that also infects mammals, birds, and reptiles. Carp edema virus, the causative agent of koi sleepy disease, is a common disease of *C. carpio* and its ornamental varieties (78). It was first detected in 1974 in Japan and then later detected in the USA in the late 1990s (78). More recently, the virus has been found in several European countries as well as in India and Iraq with mortality rates of up to 80%–100% (78). Another poxvirus of concern is salmon gill poxvirus that was first documented in Norway during the 1990s (7). Salmon gill poxvirus has been identified in farmed salmon in Scotland, Iceland, the Faroe Islands, and Canada where it causes complex gill disease with mortality rates of 70% (7), with European and Canadian strains being phylogenetically distinct (7).

### Natural composition and evolution of the fish virome

**Fig 2:**
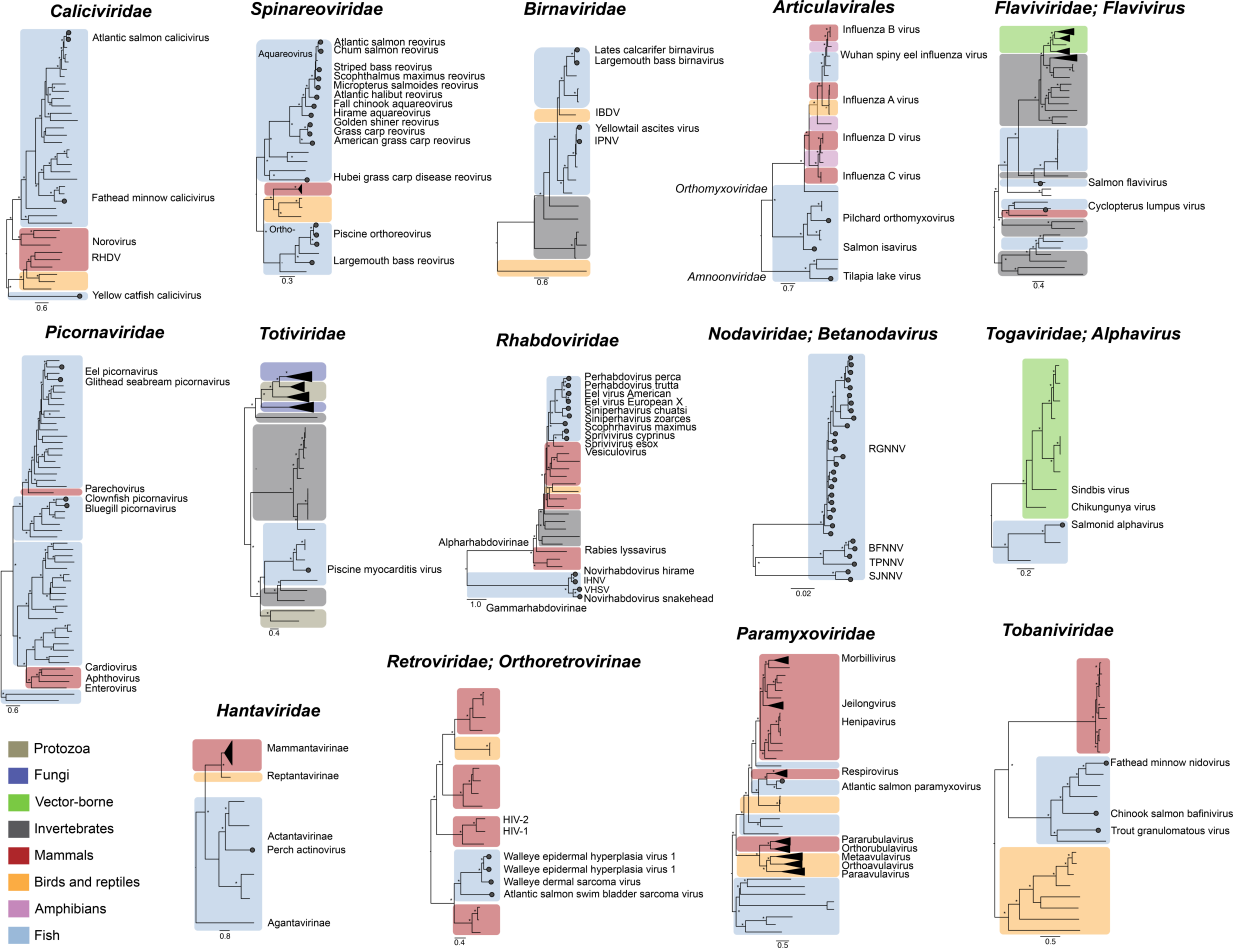
Phylogenetic relationships among notable RNA virus pathogens of fish. Maximum likelihood phylogenies were estimated using amino acid sequence alignments of the RdRp gene using MAAFT (131). The best-fit model of amino acid substitution was estimated with the “ModelFinder Plus” (-m MFP) flag in IQ-TREE (v.1.6.12) (132, 133), and 1,000 bootstrap replicates were used to estimate node support. Circles and labels on the tree tips of fish viruses denote pathogens, while all other fish viruses represent asymptomatic infections. The scale bar represents the number of amino acid substitutions per site. Asterisks denote a bootstrap value of 70% or greater.

**Fig 3:**
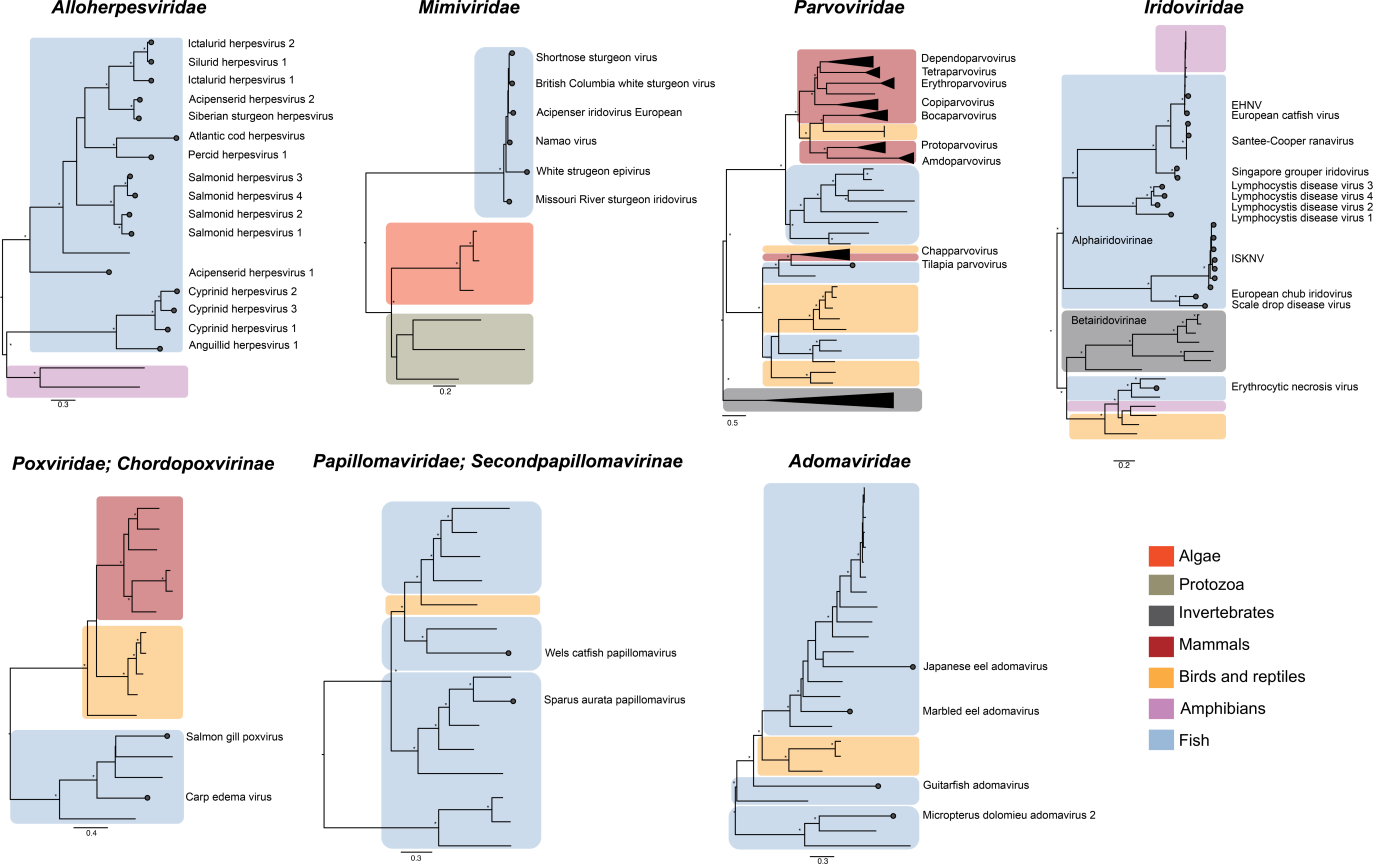
Phylogenetic relationships of notable DNA viral pathogens in fish. Maximum likelihood phylogenies were estimated using amino acid sequence alignments of the major capsid protein for the *Iridoviridae, Mimiviridae,* and *Adomaviridae*, NS1 protein for the *Parvoviridae*, E1 protein for the *Papillomaviridae*, and DNA polymerase for the *Poxviridae,* and *Alloherpesiviridae* using MAAFT (131). The best-fit model of amino acid substitution was estimated with the “ModelFinder Plus” (-m MFP) flag in IQ-TREE (v.1.6.12) (132, 133), and 1,000 bootstrap replicates were used to estimate node support. Circles and labels on the tree tips of fish viruses denote pathogens, while all other fish viruses represent asymptomatic infections. The scale bar represents the number of amino acid substitutions per site. Asterisks denote a bootstrap value of 70% or greater.

Although fish viruses were initially identified through their association with infectious disease, there is mounting evidence that in reality fish harbor diverse viral assemblages that do not result in overt disease (Fig. 2 and 3) (1–6). Indeed, RNA virus families commonly detected in healthy fish are the *Arenaviridae, Astroviridae, Bornaviridae*, *Caliciviridae, Chuviridae*, *Coronaviridae, Filoviridae, Flaviviridae* (genera *Hepacivirus* and *Flavivirus*), *Hantaviridae*, *Hepeviridae*, *Matonaviridae, Paramyxoviridae*, *Tobaniviridae, Reoviridae*, and most notably the *Picornaviridae* (1–6). Similarly, the *Poxviridae*, *Hepadnaviridae*, and *Parvoviridae* families of DNA viruses are commonly identified (2–6). It is noteworthy that most of these commensal viruses have been detected in gill tissue, and some are associated with specific organ types (1, 4). For example, fish hepaciviruses, like their mammalian counterparts (e.g., hepatitis C virus), are specifically found in liver tissue (1, 4). Similarly, viruses of the newly discovered genus *Piscichuvirus* (*Chuviridae*) are localized to the central nervous system, a feature that can be observed in both fish and reptiles (1, 4, 129, 130).

Although these viral families typically include commensal viruses of fish, they may also have the potential to emerge as disease agents. A case in point is the discovery of perch actinovirus (*Hantaviridae*) that was associated with gill disease, anorexia, and skin ulcerations in farmed European perch (*Perca fluviatilis*) (19). Also of note are members of the RNA virus order *Picornavirales* that incorporates the *Picornaviridae* and *Caliciviridae*. Notably, the *Picornaviridae* ranks among the largest and diverse families of fish viruses, with the vast majority seemingly associated with asymptomatic infections in wild populations (1, 2, 4–6). Exceptions are the genera *Potamipivirus* and *Limnipivirus* that contain pathogens including *Eel picornavirus* (Fig. 2; Table 1) (21, 75–77). Similarly, the *Caliciviridae* display extensive phylogenetic diversity of fish viruses, with only a few cases associated with emerging disease (Fig. 2; Table 1).

It is now widely accepted that the ancient evolutionary ancestors of notable human pathogens, including influenza virus (i.e., the *Orthomyxoviridae*), and SARS-CoV-2 (i.e., the *Coronaviridae*) originated in aquatic species, including fish, broadly mirroring vertebrate host evolution over a period spanning more than 500 million years (1, 3, 134, 135). Fish viruses typically form deep lineages on family-level viral phylogenies that depict a backbone of virus-host co-divergence over millions of years, albeit with regular occurrences of intra-class host-switching, including at the intra-class level (1, 3). For example, the *Hantaviridae* includes a deep jawless fish viral lineage (*Agantavirinae*) that falls basal to ray-finned fish hantaviruses (*Actantavirinae*), reptile hantaviruses (*Reptantavirinae*), and mammalian hantaviruses (*Mammantavirinae*) (Fig. 2). In addition, the *Orthomyxoviridae* contains a basal fish clade (e.g., ISAV and pilchard orthomyxovirus), and divergent fish virus lineages are found in the influenza viruses, but the overall phylogenetic pattern suggests ancient cross-species transmission events between vertebrate classes (Fig. 2) (134). A similar pattern is observed in DNA viruses of the *Chordopoxvirinae* (*Poxviridae*) (Fig. 3).

Despite the large degree of sequence divergence between viruses of fish and higher vertebrates, there are often striking similarities in the structure and function of viral structural proteins. Such similarities have been observed among the *Hepadnaviridae*, *Orthomyxoviridae*, *Paramyxoviridae*, and *Flaviviridae* (4, 136–138). For example, the neuraminidase surface glycoprotein of Wuhan spiny eel influenza virus, that phylogenetically clusters with influenza viruses (Fig. 2), exhibits sialidase activity that strongly resembles influenza B virus (1, 137). Similarly, the receptor-binding protein of several genera of the *Paramyxoviridae*, including the respiroviruses and avulaviruses—important pathogens of mammals and birds—encodes a haemagglutinin-neuraminidase (HN) domain with a β-propeller fold consisting of six anti-parallel β-sheets (i.e., “propeller blades”) (139). The second propeller blade contains a hexapeptide motif—NRKSCS—required for sialic acid binding (139). Notably, the NRKSCS sequence can be observed in salmon aquaparamyxoviruses, strongly suggesting that these viruses use similar cell-entry mechanisms to mammalian and bird paramyxoviruses. Similar observations have been noted in the structure of the African cichlid nackednavirus and hepatitis B virus capsid protein that display identical folding of the secondary-structure elements and capsid-forming core proteins (136). Nackednaviruses form a sister clade to the *Hepadnaviridae* and were estimated to have diverged ~400 million years ago (8).

It is also notable that fish viruses often cluster together on phylogenetic trees despite geographic barriers and different evolutionary pathways between fish populations. For example, Antarctic notothenioid icefishes have been restricted to the Antarctic peninsula for ~35 million years yet they harbor-related viruses (e.g., hepadnaviruses, papillomaviruses) to those found in temperate and tropical fishes including African cichlids that evolved *in situ* within the East African Great Lakes over the last 10 million years (4, 8, 9). It is likely that these viruses have been present since the early formation of the Actinopterygii (i.e., ray-finned fishes—400 million years ago) and have evolved alongside fish lineages over many millions of years.

## AQUACULTURE: A KEY CONTRIBUTOR TO THE EMERGENCE OF VIRAL DISEASES

Central to the emergence of viral diseases in aquaculture is that fish-farming practices provide a high level of connectivity between fish populations, thereby increasing interactions between donor (e.g., wildlife reservoirs) and recipient (e.g., domestic) host species. Farmed fish are often cultured in open-net cages near the shore in marine ecosystems (e.g., Atlantic salmon) or in a freshwater lake or river for inland aquaculture (e.g., rainbow trout) (140). As these animals are maintained at high population densities within the same water column as wild fish they offer ample opportunity for horizontal transmission. Open-net cages also attract wild fish species for habitat and resources, further increasing the chances of virus transmission. For example, molecular surveillance of VHSV in British Columbia, Canada, identified transmission links between wild pelagic herrings and farmed salmon through net-pen facilities (141). Similar transmission dynamics have been observed in IHNV, ISAV, IPNV, salmonid alphavirus, and nervous necrosis viruses, SJNNV, TPNNV, RGNNV, and BFNNV (140).

That viruses typically maintain infections in healthy wildlife and are often only pathogenic in farmed populations that experience stress and immunosuppression induced by overcrowding, altered nutrition and temperature changes represents a major challenge to preventing emerging viral diseases in aquaculture (142). Genetic diversity among aquaculture animals is also affected by inbreeding and selective breeding, which can result in small effective population sizes (143). In turn, the limited genetic diversity among aquaculture animals to existing physiological stressors, as well as high contact rates, will increase the likelihood of disease emergence following a species jump at the domestic-wild interface. These conditions can also facilitate the evolution of novel viral variants. For example, four lineages of IHNV circulated among rainbow trout aquaculture facilities within the same geographic region over a 20-year period (144). It was also estimated that the genetic diversification of VHSV, which exhibits extensive genetic diversity, occurred within the last 300 years, suggesting that its evolution has been heavily shaped by aquacultural practices (100).

In combination with host biological factors, the translocation of aquaculture animals (or their eggs) over vast geographic distances has caused numerous emerging viral diseases and may even introduce pathogens to wild ecosystems like coral reefs that already face threats of extinction from anthropogenic climate change. It was recently demonstrated that the emergence of PRV in wild Pacific Chinook salmon—that are on the brink of extinction—was largely shaped by the translocation of Atlantic salmon to the Pacific Ocean from Europe for aquaculture operations during the 1980s (23). In addition, the introduction of largemouth bass, which is native to North America, to China during 1983, where it now yields over 600,000 tons annually (18). Largemouth bass virus (LMBV), a ranavirus that was first detected in the Santee-Cooper lakes in the USA, has now caused disease outbreaks in farmed largemouth bass in China, with phylogenetic divergence between American and Asian strains (57). Moreover, the same LMBV strain has emerged in diseased farmed mandarin fish from China as well as healthy wrasses from the Great Barrier Reef (GBR), Australia (6, 56). Other notable examples include the movement of IHNV from North America to Europe and Asia and PMCV from Norway to the Faroe Islands from contaminated fish eggs (145, 146).

In a similar manner, the global trade of ornamental fishes has led to the emergence of novel viral pathogens. Megalocytiviruses have been associated with a wide diversity of ornamental fishes from over 17 different fish families, including cichlids (Cichlidae), platy (Poecillidae), and gouramis (Helostomatidae, Osphronemidae) (63). Notable among the megalocytiviruses is dwarf gourami iridovirus that caused disease outbreaks in various aquarium retailers across Sydney, Australia (147). A very similar virus (99.5% nucleotide sequence similarity) was associated with a large epizootic in farmed Murray cod (*Maccullochella peelii peelii*), an iconic fish in Australian waterways (147). Importantly, these disease outbreaks were linked to the importation of ornamental gouramis from Asia (148). Viral epizootics can also occur through the importation of cleaner fish and baitfishes. For example, *Cyclopterus lumpus virus* (*Flaviviridae*) was detected in diseased *C. lumpus* cleaner fish—a common biological control agent for salmon louse (*Lepeopjtjeirus salmonis*)—from a salmon aquaculture facility in western Norway (149). Subsequently, the disease spread to England with high mortality, following the importation of cleaner lumpfish from Norway (17). While *Cyclopterus lumpus virus* is seemingly specific to cleaner lumpfish, cross-species transmission of VHSV and NNV have been observed between cleaner fishes and salmon (150). Similarly, Fathead minnow nidovirus (FHMNV) (*Tobaniviridae*), which was first isolated from moribund fathead minnows (*Pimephales promelas*) from a baitfish facility, emerged in farmed muskellunge (*Esox masquinongy*) (92). High mortality was observed approximately 1 month after the diet was switched from pellets to live fathead minnow baitfish (92).

## TELEOST FISH AS A MODEL SYSTEM TO STUDY VIRUS ECOLOGY AND EVOLUTION

Teleost fishes account for more than half of the world’s vertebrate biodiversity and occupy a range of ecosystems that are found across all geographic and climate zones. From the tropics to the Antarctic, teleost fishes have evolved a broad spectrum of ecological traits both within and among ecosystems. While this diversity has been generated throughout the last 300 million years, teleost fishes also exhibit rapid rates of evolutionary change through adaptive radiations (e.g., African cichlids, Antarctic notothenioid icefishes, and three-spined stickleback fishes) (151–154). Because of their diversity, abundance, and long- and short-term evolutionary history, teleost fishes constitute a powerful and tractable model system for the study of virus ecology and evolution. In addition, teleost fishes are accessible, require low cost and maintenance, and exhibit high fecundity with often short generation times (155).

### Market fish

The utility of market fish as a model system stems from their accessibility, movement between wild and domestic environments, and natural composition of a large diversity of viruses (2, 156). For example, an analysis of 19 healthy wild-caught market fish species in Australia identified 25 novel fish viruses, including hantaviruses, filoviruses, picornaviruses, paramyxoviruses, orthoreoviruses, arenaviruses, and hepadnaviruses (2). Also of note is that the virome composition of market fishes is impacted by aspects of host biology and ecology. For example, market fish living in large shoals (e.g., garfish) carried greater viral richness than solitary fishes (e.g., flounder) (156). This indicates that high levels of contact between conspecifics within dense fish shoals—similar to what can be observed in aquacultural populations—will result in greater virus transmission than solitary fishes. Finally, as is true of many vertebrate systems, host phylogenetic relationships have played an important role in shaping fish viromes, such that there are significant differences in viral richness and communities between market fish orders (2).

### Reef fishes

The high interconnectivity among reef fishes makes them an exceptionally powerful model system to reveal the impact of host genetic diversity on virus evolution and emergence. Reef fishes account for one-third of marine fish biodiversity, exhibit diverse trophic guilds, and typically live in close proximity (6, 157). As a case in point, reef fishes from a community on the GBR were observed in densities of up to 150 fishes, representing 25 species in a sampling area of 3.5 m^2^, which was sustained over the course of a year (158). Despite their connectivity, relatively high levels of sequence divergence have been observed between viruses within reef fish communities, with very little evidence for cross-species transmission (6, 157). This indicates that high levels of genetic diversity within fish communities—reflecting the presence of fish species from many different families—should induce strong barriers to infection. In particular, genetically diverse hosts exhibit different cellular properties (e.g., receptors) that will limit virus transmission and replication (159). Indeed, with the exception of VHSV (particularly genotype IV), ISKNV, IPNV, and NNV species, most emerging viral diseases have been restricted to a single fish family (Table 1).

### African cichlids

Due to their rapid adaptive radiation of 240 species over the last 10 million years, African cichlid fishes of Lake Tanganyika constitute a remarkable system to address a wide array of questions in evolutionary biology. From a virological perspective, African cichlid fishes have been used to investigate whether and how adaptive radiations impact virus evolution and ecology, particularly in the context of cross-species transmission (4). In marked contrast to reef fishes, African cichlid viromes are characterized by high levels of cross-species transmission within Lake Tanganyika (4). A notable difference between reef fishes and African cichlids is that the cichlid radiation within Lake Tanganyika occurred very rapidly, resulting in low levels of genetic divergence between fish species (e.g., differences of less than 0.03%), while reef fishes from the GBR evolved over much longer evolutionary timescales (i.e., millions of years) with barriers set in place prior to their establishment on coral reefs within the last 10,000 years (157). In addition, as the cichlid communities in Lake Tanganyika increased in diversity ~2–3 million years ago, viruses exploited a large supply of genetically similar and susceptible hosts which, in turn, increased the rate of virus diversification. Overall, these data indicate that viruses encounter strong host barriers to infection among wild fish communities that exhibit high levels of host genetic diversity. Future research should investigate whether this phenomenon holds true for fish polycultures in aquaculture, which has been proposed as an effective system (160).

### Laboratory zebrafish

Over the last 40 years, zebrafish (*Danio rerio*) have served as an important laboratory model for the study of developmental genetics, behaviour, cancer, immunobiology, and toxicology (161). Laboratory zebrafish naturally carry picornaviruses as well as megalocytiviruses and betanodaviruses that can cause mortality (161–164). In particular, zebrafish picornavirus (ZfPV) was detected in several research institutions from North America, Europe, and Asia (161, 162). Because of its wide distribution in asymptomatic zebrafish, ZfPV has been utilized as a model to investigate transmission and infection dynamics between individuals, offering a new perspective for the study of virus-host interactions and the evolution of vertebrate immunity (162). For example, the use of a green fluorescent protein revealed picornavirus infection (and horizontal transmission) among zebrafish in parallel with the visualization of antiviral immune signaling through the expression of interferon-stimulated gene 15 (*isg15*)—a signaling protein responsible for inducing a variety of immune genes (162).

## CHALLENGES IN FISH METATRANSCRIPTOMICS AND FUTURE DIRECTIONS

While metatranscriptomic sequencing provides a powerful and unbiased analysis of viral diversity, evolution, and ecology in fish, there are several limitations. The aquatic environment supports an enormous diversity of invertebrates, algae, and single-celled eukaryotes that also contain a rich source of viruses (165, 166). Hence, a common challenge in fish viromics is distinguishing between “vertebrate-associated” viruses that infect fish or “non-vertebrate-associated” viruses that infect other organisms associated with fish diet or contamination of gill tissue. A phylogenetic approach can be utilized to help assign a virus of fish origin. For example, if a putative virus can be classified within the same genera as a *bona fide* fish virus with a proven ability to infect fish cell lines, then it is highly likely to be of fish origin. In addition, other measures such as viral abundance (e.g., the number of reads per million) and genome organization should be considered. For instance, vertebrate-associated picornaviruses are monophyletic and are clearly distinct from “picorna-like” viruses (i.e., other *Picornavirales*; 1, 2, 6, 157, 165). Picorna-like viral polyproteins are highly divergent, such that conventional bioinformatic tools often fail to detect key domains (e.g., rhv and helicase) that are found in vertebrate-associated picornaviruses. Clearly, future research should aim to improve methods that can better distinguish between vertebrate and non-vertebrate viral groups.

**Fig 4:**
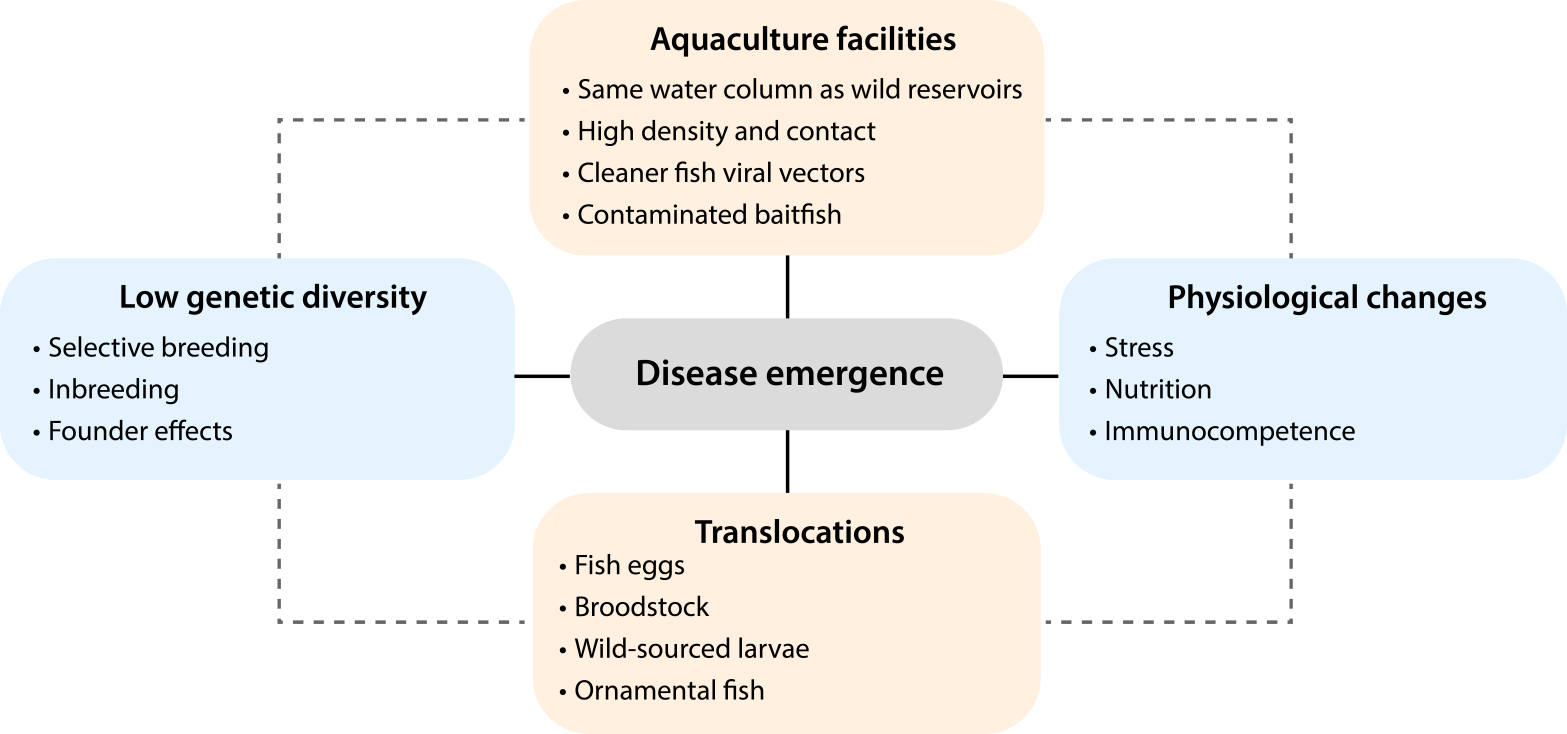
Interacting biological (blue) and ecological factors (orange) that influence viral disease emergence in aquaculture systems. Viruses are often silently translocated over vast geographic distances to new environments through the international trade of fish eggs, broodstock, wild-sourced larvae, and ornamental fishes. Viruses can enter aquaculture facilities through the international trade or through net-pen cages that attract wild reservoirs (e.g., donor hosts). Following a successful host jump—through the adaptation to a new host species—disease emergence can occur as a result of physiological changes (e.g., stress, nutrition, and immunocompetence) in the recipient host. This process can be exacerbated by low genetic diversity within dense, fish monocultures through selective breeding, inbreeding, and founder effects.

Paleoecological data predict that increasing ocean temperature and depleting oxygen concentrations will facilitate the evolution of smaller sized tropical fish species and push them poleward to temperate and polar oceans (167). Indeed, many tropical fish species are already settling on temperate reefs through a process known as “tropicalization” (168). While the general ecological impacts of tropicalization on native temperate fish populations are becoming clearer, the effects on infectious disease emergence are still uncertain. We hypothesize that viruses endemic to tropical ecosystems will increase their geographic distribution, potentially causing large-scale disease outbreaks, similar to what can be observed in aquaculture (Fig. 4). This, in turn, could be detrimental to native temperate and polar fishes, pushing them to extinction. For instance, virome studies have shown that smaller fishes (e.g., gobies, blennies, cardinalfishes) naturally carry a diverse array of viruses including those related to pathogens (e.g., parvoviruses and hantaviruses) (6, 157). Another key area of future research is understanding how virome composition changes over time and assessing whether particular viruses are more prevalent during periods of fish stress or spawning.

## CONCLUSIONS

The metagenomic revolution has redefined our understanding of viral emergence, placing it in a new ecological and evolutionary context; viruses are natural components of wild aquatic ecosystems and have co-diverged with fish over millions of years with regular cross-species transmission occurring between species despite the absence of disease. Our understanding of viral diversity in fish is advancing at a remarkable pace, making classification of these viruses challenging. While metatranscriptomic virus discovery studies are continuously revealing a multitude of novel viruses, including those of veterinary and aquacultural importance, future research should emphasize to incorporate an ecosystems approach, particularly in the context of climate change, species range shifts, and interactions at the wild-domestic interface. Hence, revealing the drivers of cross-species transmission will improve control measures against emerging viral diseases in wild and domestic aquatic ecosystems.
